# Pulmonary Embolism Masquerading as Acute Abdominal Pain: A Rare and Challenging Diagnosis

**DOI:** 10.7759/cureus.74623

**Published:** 2024-11-27

**Authors:** Fatema Jamsheer, Njood Alsudairy

**Affiliations:** 1 General Practice, Salmaniya Medical Complex, Manama, BHR; 2 Radiology, Second Health Cluster, Jeddah, SAU

**Keywords:** acute abdominal pain, anticoagulation therapy, atypical presentation, case report, ct pulmonary angiography, d-dimer, diagnostic challenge, diaphragmatic irritation, idiopathic thromboembolism, pulmonary embolism

## Abstract

Pulmonary embolism (PE) is a potentially fatal condition with variable clinical presentations, ranging from classic respiratory symptoms to rare atypical manifestations. This report describes a 47-year-old woman who presented with acute, severe right upper quadrant abdominal pain, nausea, and vomiting without respiratory complaints. Initial investigations, including abdominal ultrasound and contrast-enhanced CT of the abdomen, revealed no intra-abdominal abnormalities. Elevated D-dimer levels and incidental findings on imaging prompted further evaluation with CT pulmonary angiography, which confirmed bilateral pulmonary emboli. The patient had a history of obesity but no other identifiable thrombotic risk factors. Anticoagulation with low-molecular-weight heparin followed by rivaroxaban led to rapid symptom resolution. Comprehensive thrombophilia screening was negative, and follow-up imaging confirmed the resolution of the emboli. This case highlights the diagnostic challenges posed by PE presenting as isolated abdominal pain, an uncommon but clinically significant phenomenon. A systematic diagnostic approach, including consideration of PE in patients with unexplained abdominal symptoms and elevated D-dimer levels, is critical to avoiding delays in diagnosis and treatment. Early recognition and prompt anticoagulation therapy are essential for preventing potentially fatal outcomes and ensuring optimal patient care.

## Introduction

Pulmonary embolism (PE) is a potentially life-threatening condition resulting from the obstruction of pulmonary arteries by thromboemboli, most commonly originating from deep vein thrombosis (DVT) in the lower extremities. While classic symptoms include dyspnea, pleuritic chest pain, and tachycardia, PE is a highly variable and often elusive diagnosis due to its nonspecific clinical presentation. Atypical manifestations, such as referred pain in the abdomen, can further obscure the diagnosis and delay timely intervention [1,2].

Abdominal pain as the primary symptom of PE is rare, with few cases documented in the literature. It is hypothesized to result from diaphragmatic irritation caused by embolic involvement of pulmonary vasculature near the diaphragm. This presentation poses a diagnostic challenge, as it mimics common intra-abdominal pathologies such as cholecystitis, appendicitis, or mesenteric ischemia. Identifying such cases requires a high index of suspicion, especially when standard abdominal imaging fails to reveal a definitive cause [1-3].

This report describes a rare case of PE presenting as acute abdominal pain, emphasizing the importance of a systematic approach to diagnosis, including the use of D-dimer testing and advanced imaging. The case underscores the need for early recognition and treatment of PE to prevent potentially fatal outcomes, particularly in atypical presentations.

## Case presentation

A 47-year-old woman presented to the ED with a chief complaint of acute, severe abdominal pain. The pain had started abruptly six hours earlier, localized to the right upper quadrant, and was associated with nausea and non-bilious vomiting. She denied fever, diarrhea, melena, hematochezia, or prior episodes of similar pain. Her medical history included obesity (BMI 32 kg/m²) and a history of oral contraceptive use for dysmenorrhea, which she had discontinued three months earlier. There was no history of recent surgery, trauma, or immobilization. She denied smoking, alcohol use, or recreational drug use. Her family history was notable for a maternal uncle who had died from an undiagnosed condition presumed to involve the heart.

Upon arrival, the patient appeared in moderate distress due to pain. Her vital signs revealed tachycardia with a heart rate of 112 beats per minute, blood pressure of 128/84 mmHg, respiratory rate of 20 breaths per minute, and oxygen saturation of 96% on room air. She was afebrile. On physical examination, she had tenderness in the right upper quadrant and epigastrium without rebound tenderness or guarding. Bowel sounds were present and normal. No masses or organomegaly were palpable. Murphy's sign was negative. Cardiovascular and respiratory system examinations were unremarkable except for tachycardia. No lower limb swelling or tenderness was noted, and Homan's sign was negative.

Initial laboratory investigations showed leukocytosis (WBC count 12,400/µL), elevated D-dimer (2.1 µg/mL), normal hemoglobin and platelet count, mildly elevated liver transaminases (alanine aminotransferase 68 U/L, aspartate aminotransferase 54 U/L), and normal bilirubin levels. Renal function tests and serum amylase and lipase levels were within normal limits. Arterial blood gas analysis showed a mild respiratory alkalosis.

**Figure 1:**
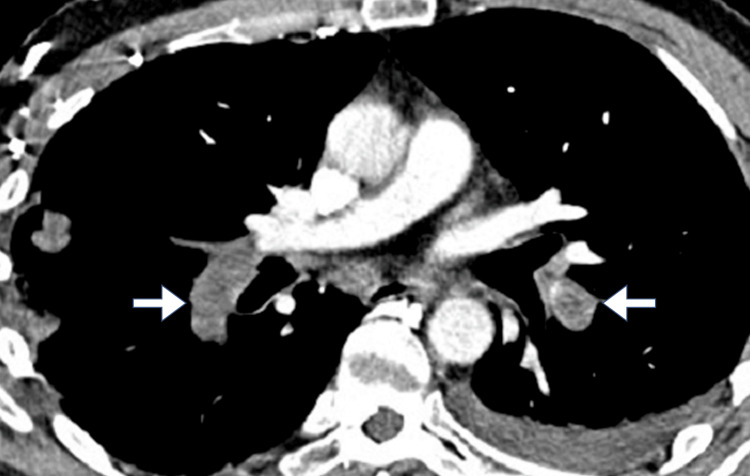
Axial CT pulmonary angiography image It demonstrates bilateral filling defects (arrow) consistent with acute pulmonary embolism.

Imaging studies included an abdominal ultrasound, which showed no evidence of gallstones, biliary dilation, or liver abnormalities. A contrast-enhanced CT scan of the abdomen and pelvis was performed to rule out intra-abdominal pathology. The scan revealed no abnormalities in the abdominal organs but incidentally noted mild bilateral basal atelectasis. Given the elevated D-dimer and the absence of clear abdominal findings, a CT pulmonary angiography (CTPA) was performed to investigate possible PE. The CTPA revealed filling defects in the segmental pulmonary arteries (Figure 1).

The differential diagnosis initially included acute cholecystitis, peptic ulcer disease, pancreatitis, mesenteric ischemia, and referred pain from thoracic causes such as PE or myocardial infarction. The abdominal findings and the patient’s clinical stability made acute cholecystitis and pancreatitis less likely. The absence of electrocardiographic or biomarker evidence ruled out myocardial infarction. The eventual diagnosis of PE explained the acute abdominal pain as referred pain from diaphragmatic irritation due to pulmonary vascular obstruction.

Management was initiated promptly with subcutaneous low-molecular-weight heparin at a therapeutic dose. The patient was admitted to the ICU for close monitoring. A comprehensive thrombophilia workup was undertaken, including tests for factor V Leiden mutation, prothrombin gene mutation, protein C and S deficiency, and antiphospholipid antibodies, which were negative. No DVT was identified on the Doppler ultrasound of the lower extremities.

The hospital course was uneventful. The patient’s abdominal pain resolved within 24 hours of initiating anticoagulation, and her vital signs normalized. She was transitioned to oral anticoagulation with rivaroxaban before discharge, with a planned treatment duration of six months. Detailed counseling was provided about the importance of medication adherence and recognizing symptoms of recurrent thrombosis.

At a one-month follow-up, the patient reported no recurrence of symptoms. Repeat imaging with a limited CTPA showed resolution of the pulmonary emboli. A multidisciplinary review concluded that the most likely etiology of her PE was idiopathic, given the absence of identifiable risk factors other than obesity. The patient continues on anticoagulation therapy with plans for re-evaluation at six months.

## Discussion

PE remains a challenging diagnosis, particularly when presenting with atypical symptoms such as acute abdominal pain. This case illustrates the diagnostic complexity associated with PE and highlights the importance of maintaining a broad differential diagnosis when evaluating patients with nonspecific abdominal pain. The unusual presentation seen here underscores the variability of PE, which is influenced by the size, location, and number of emboli, as well as patient-specific factors.

Abdominal pain in PE is rare and thought to result from diaphragmatic irritation caused by emboli in the lower pulmonary lobes or pleural-based infarction. The absence of classic respiratory symptoms, such as dyspnea or pleuritic chest pain, further complicates the clinical picture and often leads to misdiagnosis or delayed recognition [3,4]. In this case, the initial evaluation focused on abdominal etiologies, such as acute cholecystitis or pancreatitis, reflecting the diagnostic approach commonly followed in such presentations. The elevated D-dimer and incidental findings on imaging prompted further investigation, ultimately leading to the correct diagnosis of PE. This highlights the utility of D-dimer testing and the importance of incorporating it into the workup for atypical presentations despite its low specificity.

The literature documents several similar cases where PE presented as isolated abdominal pain. In one review, approximately 2-4% of patients with PE reported abdominal pain as the predominant symptom [1-4]. These cases often mimic intra-abdominal emergencies, leading to unnecessary surgical interventions or delays in appropriate management. Unlike more common causes of acute abdominal pain, the absence of localized inflammatory findings on imaging or examination should raise suspicion for extra-abdominal causes, such as PE, particularly in patients with risk factors such as obesity, hormonal therapy, or a hypercoagulable state [3-5]

A unique aspect of this case is the lack of identifiable thrombotic risk factors apart from obesity. A thorough thrombophilia workup was negative, and there was no evidence of DVT on Doppler ultrasound. This supports the diagnosis of idiopathic PE, which accounts for up to 20% of cases [3,5]. Idiopathic PE represents a diagnostic challenge, as it lacks clear precipitating factors, emphasizing the importance of early imaging when clinical suspicion is high.

From a management perspective, this case illustrates the critical role of prompt anticoagulation in treating PE, regardless of its presentation. Low-molecular-weight heparin, followed by direct oral anticoagulants, has become the standard of care, offering efficacy, safety, and ease of use compared to traditional warfarin therapy [2-4]. The resolution of symptoms within 24 hours of anticoagulation highlights the reversibility of symptoms when intervention is timely. The case also emphasizes the need for close follow-up to monitor for complications and guide decisions regarding the duration of anticoagulation therapy.

In the broader context, this case reinforces the importance of considering PE in the differential diagnosis of atypical presentations, including abdominal pain. It also highlights the need for multidisciplinary collaboration in managing complex cases, ensuring comprehensive evaluation and appropriate intervention. Comparing this case to those in the literature demonstrates that while PE with atypical presentations is uncommon, awareness and a structured diagnostic approach can prevent misdiagnosis and improve patient outcomes [1-3].

## Conclusions

This case highlights the diagnostic challenges of PE presenting atypically as acute abdominal pain, a rare but clinically significant phenomenon. It underscores the importance of maintaining a high index of suspicion for PE in patients with unexplained abdominal pain, especially when standard imaging and laboratory findings are inconclusive. Prompt use of advanced diagnostic tools, such as CTPA, and early initiation of anticoagulation are critical to preventing potentially fatal complications. This case also reinforces the value of a systematic, multidisciplinary approach to managing atypical presentations of common conditions, ultimately improving patient outcomes and expanding awareness of the diverse clinical spectrum of PE.
